# Creativity and Dementia: New Directions From Embodiment, Relationality, and Citizenship Discourse

**DOI:** 10.1093/geroni/igaa057.110

**Published:** 2020-12-16

**Authors:** Pia Kontos, Alisa Grigorovich, Romeo Colobong

**Affiliations:** The Kite Research Institute - University Health Network, Toronto, Ontario, Canada

## Abstract

There have been important advances in research on creativity that have provided a more inclusive view of everyday and ordinary creativity, including that of persons living with dementia. However, these developments are limited by a lack of engagement with scholarship on embodiment, relationality, and citizenship. We address these limitations by drawing on a relational model of citizenship that offers a critical rethinking of the nature of creativity and the imperative that these be supported in long-term dementia care. We draw on transcribed video-recorded interactions between elder-clowns and residents living with dementia in one long-term care home in central Canada. These are analyzed with reference to key theoretical tenets of the relational model of citizenship. Embodied selfhood (i.e., the primordial and socio-cultural dispositions of the body that are fundamental sources of self-expression and relationality), are identified as key to the creativity of persons living with dementia. We argue that creativity is not an individual cognitive trait but rather emerges from the complex intersection of enabling environments and the embodied intentionality of all involved. We conclude that creativity must be supported in everyday life through organizational practices and socio-political institutions that more fully support the relational, interpersonal, and affective dimensions of care.

